# Efficacy and safety of dydrogesterone monotherapy versus estradiol–dydrogesterone combination therapy in perimenopausal women: a real-world cohort study

**DOI:** 10.3389/fendo.2026.1816597

**Published:** 2026-05-08

**Authors:** Shuaiting Liu, Yuhui Zeng, Zhen Huang

**Affiliations:** Department of Gynecology, Ganzhou Women and Children’s Health Care Hospital, GanZhou, JiangXi, China

**Keywords:** dydrogesterone, estradiol, hormone therapy, kupperman menopausal index, perimenopause, real-world study

## Abstract

**Objective:**

To compare the 12-week effectiveness and safety of dydrogesterone monotherapy versus estradiol–dydrogesterone (E2/DYD) combination therapy in women with perimenopausal symptoms in a real-world clinical setting.

**Methods:**

This retrospective cohort study included 150 women treated at a tertiary gynecological outpatient clinic between July 2022 and January 2025 (dydrogesterone: n=60; E2/DYD: n=90). The primary endpoint was change in the Kupperman Menopause Index (KMI) from baseline to week 12. Repeated measures were analyzed using linear mixed-effects models. Secondary outcomes included clinical response (≥50% KMI reduction), endometrial thickness change, and adverse events. Continuous outcomes were evaluated using adjusted linear regression models, and binary outcomes were analyzed using Firth penalized logistic regression.

**Results:**

At 12 weeks, KMI decreased by 8.0 ± 6.6 points in the dydrogesterone group and 12.5 ± 7.4 points in the E2/DYD group (adjusted mean difference −4.51, 95% CI −6.90 to −2.12; p<0.001). Clinical response occurred in 31.5% and 51.2% of participants, respectively (adjusted OR 2.08, 95% CI 1.02–4.45; p=0.042). Improvements in vasomotor and mood-related symptoms were more pronounced in the combination group. Endometrial thickness increased modestly in the E2/DYD group (+0.60 mm), with week-12 mean values remaining within physiologic ranges. Any adverse event was reported in 11.1% versus 31.7% of participants (adjusted OR 3.55, 95% CI 1.29–9.77; p=0.014), predominantly mild and self-limited events.

**Conclusion:**

In this real-world cohort, E2/DYD combination therapy was associated with greater short-term symptom reduction compared with dydrogesterone monotherapy. Although adverse events were more frequent with combination therapy, they were generally mild and clinically manageable, and no concerning endometrial safety signals were observed over 12 weeks. As a retrospective observational study, causal conclusions cannot be drawn, and these findings should be regarded as hypothesis-generating. They provide real-world contextual evidence to inform clinical discussions around hormone therapy selection in perimenopausal care, pending confirmation in prospective randomized trials.

## 1 Introduction

The perimenopausal transition is characterized by fluctuating ovarian steroidogenesis and neuroendocrine instability resulting from progressive follicular depletion. These hormonal changes give rise to heterogeneous vasomotor, somatic, and psychoaffective symptoms that may persist for prolonged periods and significantly impair health-related quality of life. With the global population ageing and the number of women aged ≥50 years projected to increase substantially, effective and pragmatic symptom management has become an important public health priority (1–3).

Hormone therapy (HT) remains the most effective treatment for moderate-to-severe vasomotor symptoms and is recommended by major international guidelines for appropriately selected women (4, 5). In women with an intact uterus, systemic estrogen requires adequate progestogenic opposition to ensure endometrial protection. However, progestogens are not pharmacologically equivalent; differences in receptor selectivity and downstream metabolic, cardiovascular, and breast tissue effects may influence tolerability and overall risk–benefit balance (6). Dydrogesterone (DYD), a selective retroprogesterone with minimal androgenic or glucocorticoid activity, has demonstrated effective endometrial protection when combined with estrogen and is generally regarded as having a favorable safety profile compared with older synthetic progestogens (7, 8).

In perimenopausal women with residual ovarian activity, progestogen monotherapy is sometimes prescribed to address irregular bleeding, sleep disturbance, or mood symptoms, potentially mediated through progesterone-sensitive neuroactive pathways (9). Nevertheless, because vasomotor instability is closely linked to estrogen deficiency, monotherapy may be insufficient in women with prominent vasomotor symptoms. Despite widespread clinical use of both strategies, direct comparative real-world evidence between dydrogesterone monotherapy and estradiol–dydrogesterone (E2/DYD) combination therapy remains limited. We therefore conducted a retrospective cohort study of 150 perimenopausal or recently postmenopausal women to compare 12-week changes in the Kupperman Menopause Index (KMI) and short-term safety outcomes between these two therapeutic approaches.

## 2 Methods

### 2.1 Study design and setting

This retrospective real-world cohort study was conducted at a tertiary-level gynecological endocrinology outpatient clinic. We systematically reviewed electronic medical records of consecutively registered perimenopausal and early postmenopausal women who initiated either dydrogesterone (DYD) monotherapy or estradiol–dydrogesterone (E2/DYD) combination therapy between July 2022 and January 2025. The index date was defined as the first prescription date for the eligible regimen during the study period. Follow-up assessments were scheduled at baseline (T0), week 4 (T1), week 8 (T2), and week 12 (T3) according to routine clinical practice.

The study protocol was approved by the Medical Ethics Committee of Ganzhou Maternal and Child Health Hospital (Approval No [2025].伦审临第(19)号). The requirement for individual informed consent was waived due to the retrospective nature of the study and the use of de-identified data. All data were anonymized prior to analysis. The study was conducted in accordance with the Declaration of Helsinki and reported in line with the STROBE statement for observational studies. As a retrospective observational analysis, this study cannot establish causal relationships between treatment allocation and clinical outcomes. The observed associations may be subject to residual confounding from variables not captured in the medical record, including prior hormonal therapy use, tobacco use, and psychiatric comorbidity severity, and should not be interpreted as evidence of therapeutic superiority.

### 2.2 Participants

Women aged 40–60 years presenting with perimenopausal or early postmenopausal symptoms and receiving a new prescription for either DYD monotherapy (10 mg twice daily) or oral E2–1 mg/DYD 10 mg combination therapy during the recruitment period were eligible.

Menopausal stage was defined as follows: perimenopause was identified by irregular menstrual cycles or amenorrhoea lasting <12 months, accompanied by a serum follicle-stimulating hormone (FSH) level >25 IU/L; early postmenopause was defined as amenorrhoea ≥12 months. To enhance internal validity, women were required to have baseline symptom assessment and transvaginal ultrasonography available within the predefined baseline window (e.g., within 2 weeks of treatment initiation).

Exclusion criteria included: (1) absolute contraindications to systemic hormone therapy, including unexplained genital bleeding, known or suspected estrogen-dependent malignancy, active or recent thromboembolism, and severe hepatic impairment; (2) use of other hormonal preparations within the preceding 3 months; (3) baseline endometrial thickness >10 mm; and (4) incomplete baseline documentation precluding primary outcome evaluation.

A total of 178 women were screened; 28 were excluded (incomplete records, n=11; not meeting eligibility criteria, n=12; declined inclusion in the institutional registry, n=5), resulting in a final analytic cohort of 150 women (DYD: n=60; E2/DYD: n=90). The 1:1.5 allocation ratio reflected prevailing prescribing patterns at the study site rather than investigator-driven assignment.

### 2.3 Outcome measures

The primary outcome was the change in Kupperman Menopause Index (KMI) total score from baseline (T0) to week 12 (T3). The KMI is a composite symptom instrument consisting of 11 domains with weighted scoring (maximum 51 points), where higher scores indicate greater symptom severity (10).

Key secondary symptom outcomes were pre-specified hot flush, insomnia, and mood disturbance domains because of their clinical relevance and sensitivity to hormonal modulation. Secondary endpoints included: (1) clinical response at week 12, defined *a priori* as ≥50% reduction in KMI total score from baseline; (2) longitudinal KMI trajectory across T0–T3; (3) change in KMI symptom domains over follow-up; (4) endometrial thickness measured by transvaginal ultrasonography at baseline and week 12; (5) incidence of pre-specified adverse events (vaginal bleeding, breast tenderness, nausea, and any adverse event); and (6) treatment adherence.

Endometrial thickness was assessed using transvaginal ultrasonography as part of routine care, recorded in millimeters as the maximal double-layer thickness in the sagittal plane. Adverse events were abstracted from clinical notes and follow-up visit documentation; events were adjudicated as present if explicitly recorded by clinicians during follow-up. Treatment adherence was estimated from prescription refill records and patient-reported adherence at follow-up visits. A structured review of clinical records was conducted to identify any documented dose modifications, treatment interruptions exceeding seven consecutive days, or switches to a different hormonal preparation during the 12-week observation period. No such events were documented in the medical records of any complete-case participant.

### 2.4 Statistical analysis

Baseline characteristics are summarized as mean ± SD for continuous variables and counts (percentages) for categorical variables. Between-group comparisons were performed using Welch’s t-test for continuous variables and χ² or Fisher’s exact test for categorical variables, as appropriate.

For the primary endpoint, the main analysis was conducted using complete-case data, consistent with the routine follow-up structure of this real-world cohort (available cases: n=136; DYD n=54; E2/DYD n=82). Missingness was assessed by comparing all available baseline characteristics between patients with and without documented 12-week visits; no statistically significant differences were identified (all p>0.10), providing limited empirical reassurance regarding the comparability of observed baseline characteristics, but not establishing the missing-at-random assumption. This assumption cannot be definitively verified for unmeasured dimensions and is acknowledged as a limitation. The potential impact of complete-case exclusion on the study estimates warrants explicit consideration. If the 14 patients without documented 12-week visits were disproportionately those who discontinued therapy early due to adverse events or poor tolerability, their exclusion would inflate the observed treatment response, particularly in the E2/DYD group, which carried a higher adverse event burden. Conversely, if non-attendance reflected early symptom resolution and consequent disengagement from follow-up, the complete-case analysis may underestimate the true treatment-associated difference. Because the underlying reason for non-attendance could not be ascertained from the medical record, the direction of any resultant bias remains indeterminate, and the primary estimates should be interpreted with this uncertainty in mind. No *a priori* sample size calculation was performed given the retrospective design. *Post-hoc* power analysis for the primary outcome (n_1_=54, n_2_=82; Cohen’s d=0.63; two-sided α=0.05) estimated approximately 95% power for the primary comparison. Secondary binary outcomes were not individually powered; non-significant results should be interpreted with caution as potentially reflecting insufficient statistical precision.

Longitudinal KMI measurements across T0–T3 were analyzed using a linear mixed-effects model (LMM) with fixed effects for time (categorical or per-interval), treatment group, and the time × group interaction, and a random intercept for participant to account for within-subject correlation. This framework estimates differential trajectories between regimens and is robust to unbalanced repeated measures under a missing-at-random assumption. The modelling approach was aligned with published methodological examples for repeated symptom measures in real-world cohorts (11).

Given the statistically significant baseline imbalance in mood disturbance score (p=0.005), prespecified adjusted analyses incorporated baseline mood score as a covariate in the primary models. For binary outcomes (e.g., clinical response and adverse events), effect sizes are reported as odds ratios (ORs) with 95% confidence intervals. Firth penalized logistic regression was used for binary endpoints to mitigate small-sample bias and handle separation due to rare events (e.g., zero cells). Continuous week-12 outcomes (e.g., KMI change, endometrial thickness change) were analyzed using linear regression models, with adjustment for key baseline covariates (age, BMI, and baseline mood score), and results are presented as adjusted mean differences (MDs) with 95% confidence intervals.

All tests were two-sided with a significance threshold of p<0.05. Analyses were conducted using R (version 4.3.3) (12).

## 3 Results

### 3.1 Patient flow and baseline characteristics

**Figure 1:**
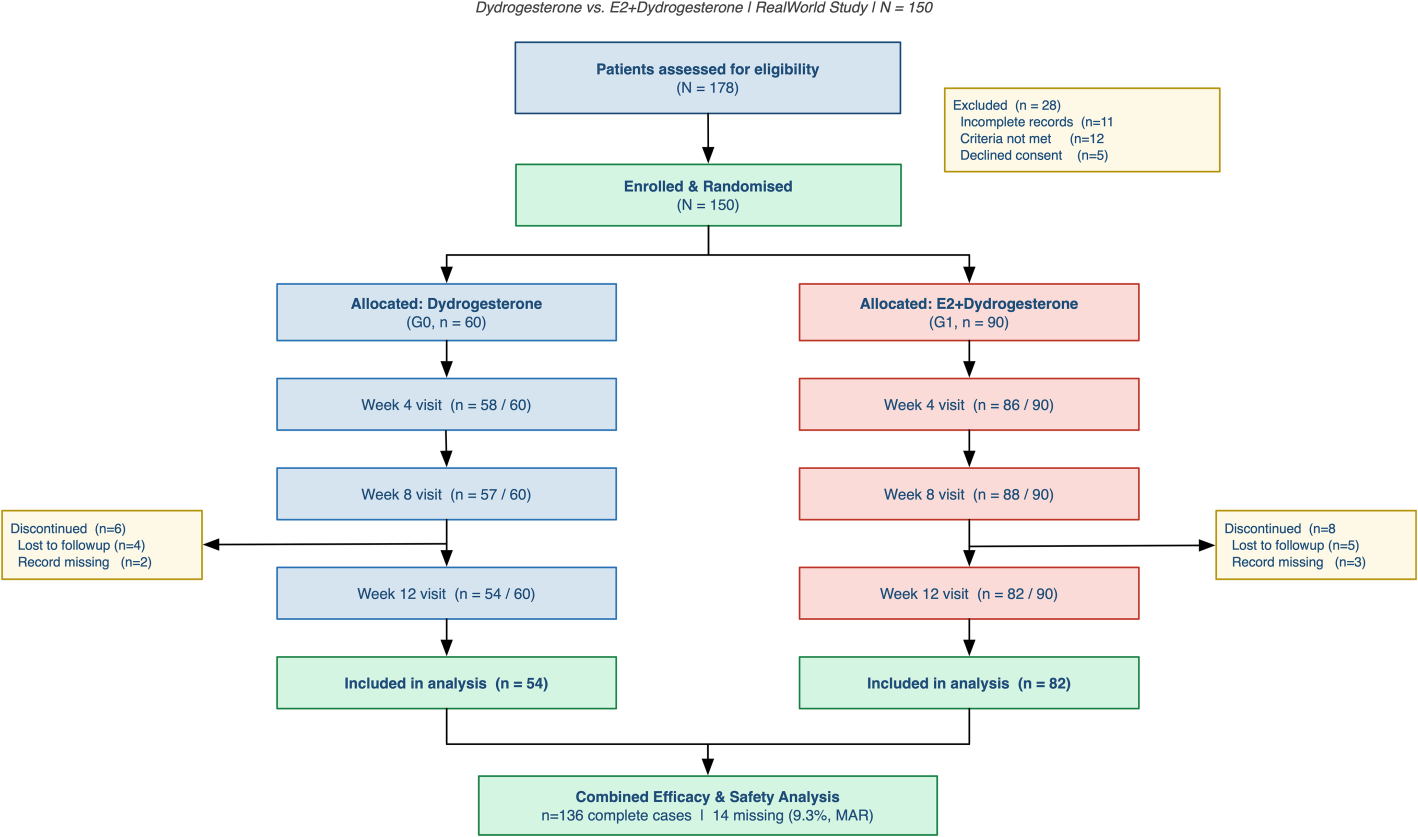
Study flow diagram.

Of 178 women screened, 150 were enrolled (G0 n=60, G1 n=90; **Figure 1**). Fourteen patients (9.3%) had no documented 12-week visit in the electronic medical record, leaving 136 complete-case participants (G0 n=54, G1 n=82). A comparison of all available baseline characteristics between patients with and without 12-week documentation identified no statistically significant differences on any measured variable (all p>0.10), providing limited empirical reassurance regarding the comparability of observed baseline characteristics, but not establishing the missing-at-random assumption; informative missingness on unmeasured dimensions cannot be excluded. As shown in **Table 1**, the two groups were well balanced at baseline. Mean age was 47.3 ± 3.2 years (G0) and 48.3 ± 3.0 years (G1; p=0.052); BMI, menopausal status, parity, comorbidity burden, education level, symptom duration, and endometrial thickness were all comparable (all p>0.13). The only significant imbalance was in the mood disturbances KMI subitem (G0: 1.2 ± 0.9 vs G1: 1.7 ± 1.1; p=0.005), which was therefore included as a covariate in all adjusted outcome models. A formal interaction test between menopausal status and treatment group in the linear mixed-effects model yielded p=0.41, providing no evidence of significant effect modification by menopausal stage, though this test was not adequately powered to detect clinically meaningful interactions. No dose modifications, treatment interruptions, or therapy switches were documented in the medical records of any complete-case participant during the 12-week observation period.

**Table 1:** Baseline characteristics of the study population.

Variable	Dydrogesterone (n=60)	E2+dydrogesterone (n=90)	P value
Age (years)	47.3 ± 3.2	48.3 ± 3.0	0.052
BMI (kg/m²)	23.3 ± 2.9	23.5 ± 2.2	0.602
Education level, n (%)	0.198		
• Primary school	18 (30.0%)	17 (18.9%)	
• Secondary school	20 (33.3%)	41 (45.6%)	
• University or above	22 (36.7%)	32 (35.6%)	
Parity	1.7 ± 0.8	1.8 ± 0.7	0.227
≥1 Comorbidity, n (%)	23 (38.3%)	28 (31.1%)	0.256
Postmenopausal status, n (%)	29 (48.3%)	46 (51.1%)	0.868
Symptom duration (months)	9.8 ± 6.6	11.5 ± 7.0	0.131
KMI total score (baseline)	24.8 ± 6.2	26.2 ± 6.0	0.151
• Hot flush subitem	7.1 ± 3.7	7.4 ± 3.1	0.614
• Insomnia subitem	1.8 ± 1.0	1.6 ± 1.1	0.377
• Mood disturbances subitem	1.2 ± 0.9	1.7 ± 1.1	**0.005**
Endometrial thickness (mm)	6.1 ± 1.7	6.2 ± 1.6	0.764
Treatment adherence (%)	83.3 ± 11.6	85.5 ± 11.1	0.249

Continuous variables are presented as mean ± SD and compared using two-sample t-tests. Categorical variables are presented as n (%) and compared using χ² or Fisher’s exact test as appropriate. Baseline mood score imbalance (p=0.005) was included as a covariate in adjusted outcome models. Bold values indicate statistically significant between-group differences (p<0.05).

### 3.2 Primary outcome: KMI total score change

**Figure 2:**
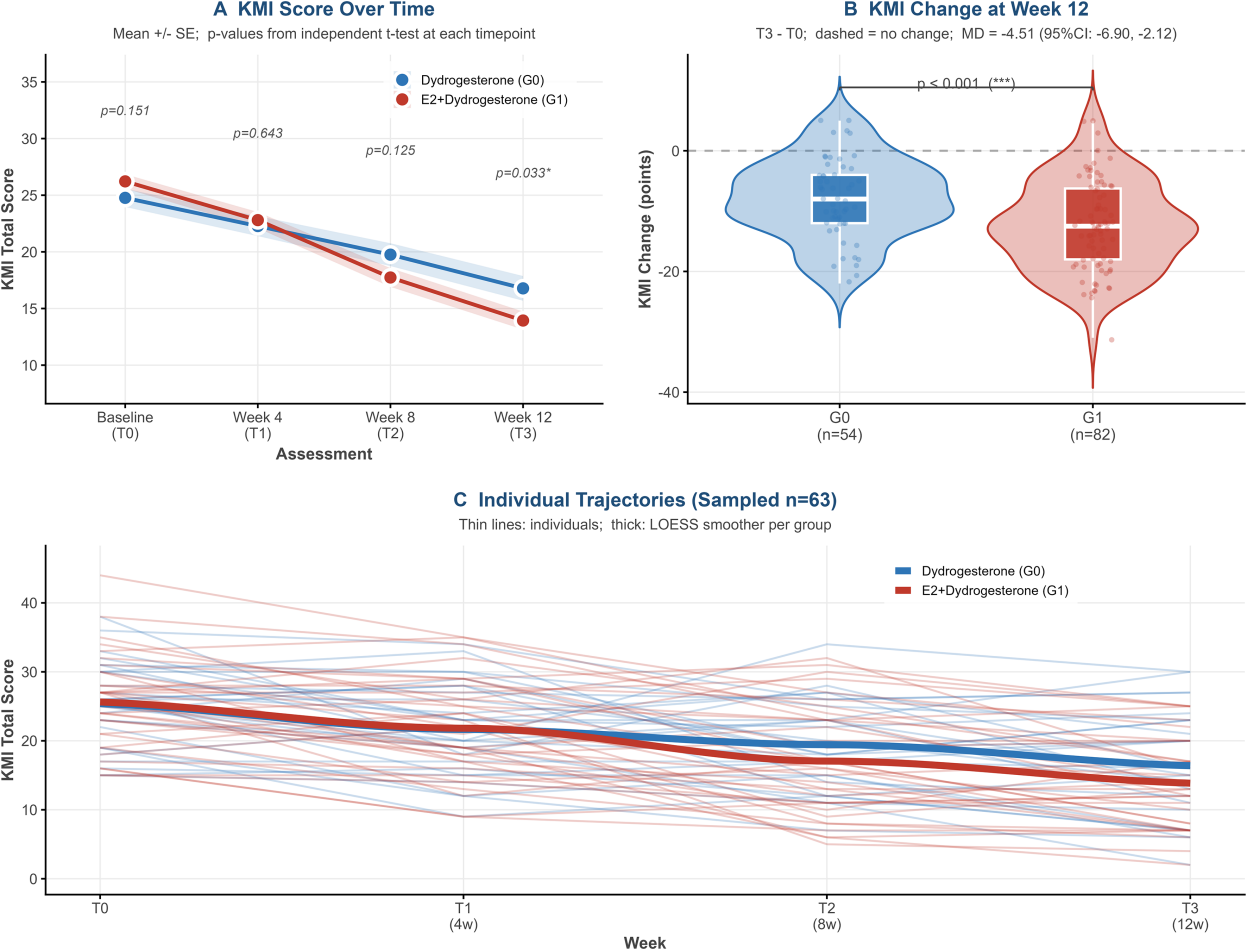
Kupperman menopause index (KMI) trajectory and treatment response over 12 weeks. **(A)** KMI score over time. Mean+/- SE; p-values from independent t-test at ewach timepoint. **(B)** KMI change at week 12. T3 - T0; dashed, no change; MD = -4.51 (95%Cl: -6.90, -2.12); ***p < 0.001. **(C)** Individual trajectories (sampled n=63). Thin lines, individuals; thick, LOESS smoother per group.

E2+dydrogesterone produced significantly greater KMI reduction over 12 weeks: −12.5 ± 7.4 points in G1 versus −8.0 ± 6.6 points in G0 (adjusted MD −4.51, 95% CI −6.90 to −2.12; p<0.001; Cohen’s d=0.63; **Table 2**). Both values exceed the KMI minimal clinically important difference of 3–4 points, and the proportional reductions were 47.7% (G1) and 35.9% (G0). The temporal trajectory (**Figure 2A**) is informative: at week 4, scores were nearly identical (G1: 22.8 ± 6.6 vs G0: 22.3 ± 6.7; p=0.643), consistent with the expected delayed clinical response to estrogen-containing therapy; by week 8 G1 pulled ahead (17.7 ± 7.7 vs 19.8 ± 7.6; p=0.125), reaching statistical significance at week 12 (13.9 ± 7.2 vs 16.8 ± 8.0; p=0.033). The distribution of week-12 KMI change is shown in **Figure 2B**. The net trajectory shift—from a baseline gap of +1.5 points (G1 higher) to −2.8 points at week 12—corresponds to the significant time×group interaction in the LMM (p=0.018) and is visually evident in the individual LOESS trajectories (**Figure 2C**). Clinical response (≥50% KMI reduction) was 51.2% in G1 versus 31.5% in G0 (adjusted OR 2.08, 95% CI 1.02–4.45; p=0.042; NNT = 5.1), well within published reference ranges for combined versus progestogen-only HT.

**Table 2:** Clinical efficacy and safety outcomes at 12 weeks (complete-case analysis).

Outcome	Dydrogesterone	E2+dydrogesterone	Adjusted effect (95% CI)	P value
KMI total score change (ΔT3–T0)	−8.0 ± 6.6	−12.5 ± 7.4	MD = −4.51 (−6.90, −2.12)	<0.001
Clinical response (≥50% reduction)	17 (31.5%)	42 (51.2%)	OR = 2.08 (1.02, 4.45)	0.042
Endometrial thickness change (mm)	−0.05 ± 0.77	+0.60 ± 0.91	MD = +0.65 (0.37, 0.93)	<0.001
Vaginal bleeding	5 (9.3%)	16 (19.5%)	OR = 2.24 (0.73, 6.91)	0.146
Breast tenderness	0 (0.0%)	8 (9.8%)	OR = 6.31 (0.76, 52.3)	0.089
Nausea/GI symptoms	2 (3.7%)	4 (4.9%)	OR = 1.33 (0.23, 7.63)	0.747
Any adverse event	6 (11.1%)	26 (31.7%)	OR = 3.55 (1.29, 9.77)	0.014

Continuous outcomes were analyzed using linear regression adjusted for age, BMI, and baseline mood score. Binary outcomes were analyzed using Firth penalized logistic regression with the same covariate adjustment. Primary analysis was conducted on complete cases (n=54 vs n=82).

### 3.3 KMI subscale changes

**Figure 3:**
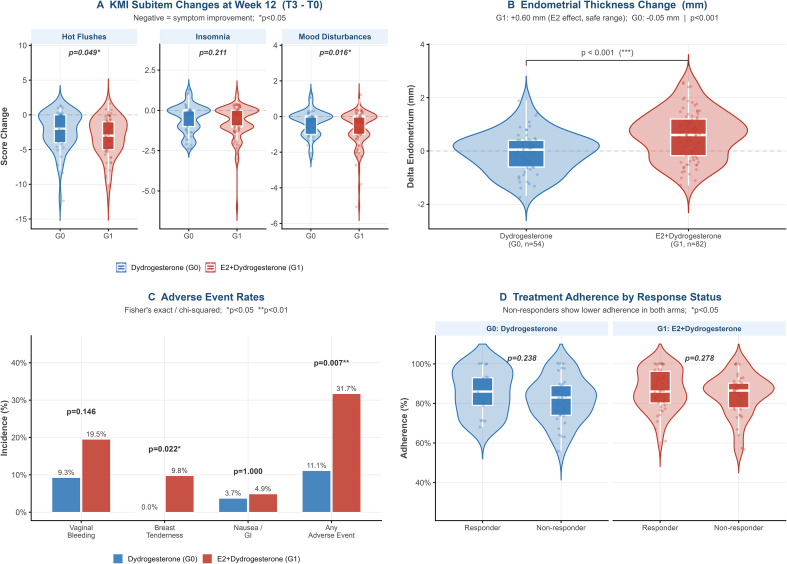
Secondary outcomes including symptom subitems, endometrial thickness, adverse events, and treatment adherence. **(A)** KMI subhite, changes at week 12 (T3 - T0). Negative, symptom improvement; *p<0.05. **(B)** Endometrial thickness change (..). G1, +0.60 mm (E2 effect, safe range); G0, -0.05 mm | ***p< 0.001. **(C)** Adverse events rates. Fisher's exact / chi-squared; *p<0.05, **p<0.01. **(D)** Treatment adherence by response status. Non-responders show lower adherence in both arms; *p<0.05.

Hot flush scores fell more in G1 (−3.18 ± 2.83 vs −2.22 ± 2.65; p=0.049; **Figure 3A**), consistent with estrogen’s direct restoration of hypothalamic thermoregulatory tone. Mood disturbance improved more in G1 (−0.71 ± 0.99 vs −0.33 ± 0.67; p=0.016), likely reflecting synergistic estrogen–neurosteroid activity on the GABA-A axis; given the baseline mood imbalance, this finding is interpreted with appropriate caution pending further sensitivity analysis. Insomnia improvement was non-significantly greater in G1 (−0.73 ± 1.03 vs −0.52 ± 0.86; p=0.211), plausibly because the low baseline insomnia burden in both arms (1.6–1.8 points) was already adequately addressed by dydrogesterone’s neuroactive metabolite 20α-dihydrodydrogesterone, leaving limited incremental benefit for the estrogen component.

### 3.4 Endometrial safety

G0 endometrial thickness was essentially stable at week 12 (−0.05 ± 0.77 mm; mean 6.0 ± 1.8 mm), confirming no estrogenic stimulation with monotherapy (**Figure 3B**). G1 showed a modest but significant increase (+0.60 ± 0.91 mm; adjusted MD + 0.65, 95% CI 0.37–0.93; p<0.001; week-12 mean 6.8 ± 2.0 mm), which is consistent with the endometrial safety profile established for E2/DYD in controlled trials. Importantly, only 4.9% of G1 patients exceeded the 10 mm surveillance threshold, and no patient in either group required endometrial biopsy or further investigation, indicating that no clinically concerning endometrial findings were identified within the 12-week observation window. The 12-week follow-up duration is insufficient to evaluate longer-term endometrial safety, which conventionally requires surveillance over at least 12–24 months; these short-term findings should not be interpreted as evidence of long-term endometrial protection.

### 3.5 Adverse events

Any adverse event occurred in 31.7% of G1 versus 11.1% of G0 patients (adjusted OR 3.55, 95% CI 1.29–9.77; p=0.014) (**Figure 3C**). All events were mild-to-moderate and no patient discontinued treatment. Vaginal bleeding was more frequent in G1 (19.5% vs 9.3%; OR 2.24; p=0.146), representing expected estrogen-driven endometrial withdrawal bleeding rather than pathology. Breast tenderness occurred exclusively in G1 (9.8%; OR 6.31; p=0.089), well below the >20% incidence threshold associated with mammographic density changes, and consistent with transient estrogenic stromal stimulation. Nausea was infrequent and equivalent (G0 3.7% vs G1 4.9%; p=0.747), confirming that dydrogesterone itself is well tolerated in both regimens.

### 3.6 Treatment adherence and responder analysis

Adherence was high and equivalent across both arms (G0: 83.3 ± 11.6%; G1: 85.5 ± 11.1%; p=0.249), confirming that the higher adverse event burden in G1 did not impair treatment-taking behavior (**Figure 3D**). Within each arm, responders showed numerically higher adherence than non-responders (G0: 85.7% vs 81.7%, p=0.235; G1: 86.8% vs 84.2%, p=0.278), a consistent though non-significant trend suggesting a dose-exposure effect. The adherence gap was wider in G0 (4.0 vs 2.6 percentage points in G1), which may reflect greater dependence of progestogen monotherapy on consistent dosing to sustain threshold neuroactive metabolite concentrations, whereas the estrogen component in G1 may buffer occasional missed doses through maintained hypothalamic stabilization.

## 4 Discussion

The principal finding of this retrospective cohort study is that E2/DYD combination therapy was associated with significantly greater reductions in perimenopausal symptom burden compared with dydrogesterone monotherapy, as measured by KMI total score change at 12 weeks. The treatment-associated difference of −4.51 points exceeds a clinically meaningful threshold, as both groups demonstrated reductions surpassing the severity category boundary of 15 points commonly used to define mild symptom status (10). This was corroborated by a higher clinical response rate (51.2% vs 31.5%), greater improvements in hot flush and mood disturbance subscores, and a significant time × group interaction in the LMM indicating progressive trajectory divergence. As a retrospective observational study, these associations may reflect residual confounding, including confounding by indication, and should not be interpreted as evidence of causal superiority. Confounding by indication is a particularly salient concern in this non-randomized setting. Prescribers may have systematically allocated E2/DYD to women presenting with more prominent vasomotor symptoms or greater overall symptom burden, while reserving dydrogesterone monotherapy for those with milder symptoms, residual ovarian activity, or a predominantly neuropsychological symptom profile. Such differential allocation based on symptom character — even when aggregate KMI total scores appear balanced at baseline, as in **Table 1** — could independently inflate the observed between-group difference in favor of E2/DYD. Because the clinical rationale underlying each prescribing decision was not formally documented in the electronic medical record, this source of confounding cannot be quantified or adjusted for, and represents a fundamental interpretive limitation of the present study. The observed associations should accordingly be regarded as hypothesis-generating, and replication in prospective designs with protocol-driven allocation is required before the observed differences can be attributed to treatment.

### 4.1 Mechanistic context for the observed symptom difference

The greater symptom reduction observed with E2/DYD relative to dydrogesterone monotherapy is biologically plausible, though mechanistic conclusions cannot be drawn from clinical symptom scores in an observational study. Vasomotor symptoms of perimenopause are primarily driven by estrogen withdrawal, which destabilizes hypothalamic thermoregulation through dysregulation of kisspeptin–neurokinin B (KNDy) neuron signaling and downstream neuroendocrine pathways (15). Estrogen supplementation directly addresses this central pathway, consistent with the significantly greater hot flush score improvements in G1 (16). The mild but consistent improvement in G0 patients is likely attributable to the anxiolytic and hypnotic properties mediated by dydrogesterone’s ability to increase brain allopregnanolone levels — a neuroactive steroid that acts as a positive allosteric modulator of GABA−A receptors (9). The synergistic interaction between estrogen-mediated central stabilization and DYD-derived neurosteroid activity in G1 likely accounts for the proportionally larger mood disturbance advantage relative to the hot flush advantage.

The delayed emergence of significant between-group KMI differences — non-significant at 4 weeks, significant at 12 weeks — is consistent with clinical observations that neuroendocrine stabilization following hormone therapy initiation may require several weeks to manifest as symptom improvement (13, 19). This temporal pattern has important clinical implications, as premature treatment modification based on insufficient response at 4 weeks would underestimate the eventual therapeutic benefit of E2/DYD therapy.

### 4.2 Endometrial safety and dydrogesterone’s protective role

The modest endometrial thickness increase in G1 (+0.60 mm) fell within the +0.3 to +0.8 mm consistent with the endometrial safety profile demonstrated in controlled trials of E2/DYD formulations (17), and mean week-12 thickness (6.8 mm) remained well below the 10 mm surveillance threshold. Only 4.9% of G1 patients exceeded this threshold, and no patient required invasive endometrial investigation. These short-term observations are consistent with — but cannot confirm — the endometrial safety profile of DYD-containing combined HT documented in longer-term studies and recent reviews (18, 20). The 12-week observation period does not permit conclusions regarding long-term endometrial risk; extended surveillance would be required. The receptor selectivity and metabolically neutral profile of dydrogesterone relative to older synthetic progestogens may contribute to these observations, though causal inference from observational data is not warranted.

### 4.3 Adverse events and breast safety

The higher overall adverse event rate in G1 (31.7% vs 11.1%; adjusted OR 3.55, 95% CI 1.29–9.77; p=0.014) warrants contextualization. The most substantive contributor was vaginal bleeding (19.5% vs 9.3%), which represents an expected estrogenic effect in peri- and early postmenopausal women undergoing cyclical stimulation of the endometrium. Breast tenderness was recorded exclusively in G1 (9.8%; adjusted OR 6.31, 95% CI 0.76–52.3; p=0.089). Crucially, the 9.8% breast tenderness incidence did not result in treatment discontinuation. Breast density and longer-term mammographic effects cannot be evaluated from 12-week clinical symptom data and should not be inferred from this finding. Longer-term breast safety evaluation, including the relationship between progestogen type and breast cancer risk (8, 21), was beyond the scope of this 12-week study. All adverse events were mild-to-moderate in severity and all patients continued their assigned therapy. These findings should be interpreted within a benefit-risk framework. The greater symptom reduction associated with E2/DYD — including a higher clinical response rate (NNT ≈ 5) and a clinically meaningful KMI score advantage — must be weighed against an adverse event burden that was approximately three times higher than that observed with dydrogesterone monotherapy. Given that all adverse events were mild, physiologically anticipated, and self-limiting, this trade-off may be acceptable for women with moderate-to-severe vasomotor symptoms in whom estrogen is not contraindicated; however, individual tolerability, patient preferences, and comorbidity profile should guide shared clinical decision-making. Of clinical importance, the higher adverse event rate in G1 did not translate into measurable impairment of adherence within this closely monitored cohort (G0: 83.3 ± 11.6% vs G1: 85.5 ± 11.1%; p=0.249). Nevertheless, in less structured real-world settings, symptoms such as vaginal bleeding and breast tenderness carry a recognized risk of prompting unsupervised treatment discontinuation, which would attenuate the symptomatic benefit of E2/DYD. Proactive patient counselling regarding the expected and transient nature of these estrogen-related effects is therefore an important component of clinical management when initiating combination therapy.

### 4.4 Clinical implications

In this retrospective cohort, E2/DYD combination therapy was associated with a higher clinical response rate and a clinically meaningful symptom score difference compared with dydrogesterone monotherapy. The estimated number of patients who would need to receive E2/DYD rather than monotherapy to achieve one additional clinical responder was approximately 5. However, given the non-randomized design and potential for confounding by indication, these findings should be regarded as hypothesis-generating rather than as evidence of therapeutic superiority. These observations are consistent with current guideline recommendations that estrogen-containing regimens address vasomotor symptoms more comprehensively than progestogen-only approaches, and may inform clinical discussions in patients without estrogen contraindications. Treatment selection should be guided by individual patient characteristics, risk factors, and evidence from prospective randomized trials (14). Dydrogesterone monotherapy remains a clinically appropriate option for women with estrogen contraindications or predominantly neuropsychological symptom profiles.

The temporal trajectory of response supports a minimum observation period of 8–12 weeks before treatment modification is considered, as meaningful between-group differences only emerged after the 4-week assessment. The high adherence rates observed in both groups (>83%) suggest that both regimens are acceptable to patients in routine clinical practice.

### 4.5 Future research directions

The present findings identify several priorities for future research. First, prospective randomized controlled trials directly comparing dydrogesterone monotherapy with E2/DYD combination therapy are needed to establish whether the observed efficacy differences reflect true treatment effects, free from the selection biases and unmeasured confounding inherent in observational cohort designs. Such trials should incorporate pre-specified primary and secondary endpoints, patient-reported outcomes, and intention-to-treat analyses. Second, extended follow-up of at least 12–24 months is required to characterize the longer-term endometrial and breast safety profiles of both regimens, consistent with the surveillance periods employed in regulatory trials of hormone therapy. The current 12-week observation window is insufficient to draw conclusions regarding cumulative endometrial or mammographic risk. Third, larger multicentre cohort studies would enable adequately powered subgroup analyses by menopausal stage, baseline symptom severity, and age, and would improve the generalizability of findings beyond a single tertiary center. Fourth, future real-world studies should systematically capture key confounding variables — including prior hormonal therapy use, tobacco use, physical activity, body composition, and comorbid mood disorders — and apply propensity score-based or other causal inference methods to strengthen the credibility of observational treatment comparisons. Collectively, such research would provide the evidence base required to support individualized, guideline-concordant hormone therapy selection in perimenopausal care.

### 4.6 Limitations

The retrospective observational design precludes causal inference. The 1:1.5 treatment allocation ratio reflected prevailing prescribing patterns rather than randomization, introducing the risk of selection bias and confounding by indication: women with more prominent vasomotor symptoms may have been preferentially prescribed E2/DYD, which may bias the observed treatment difference, although the direction and magnitude of this bias cannot be determined from the available data. Key confounders including prior hormonal therapy use, tobacco use, alcohol intake, physical activity, and psychiatric comorbidity severity were not systematically recorded in the electronic medical record and could not be controlled. Adjusted models incorporated only age, BMI, and baseline mood score, and residual confounding from unmeasured variables — including prior contraceptive use, body composition, tobacco use, and co-morbid mood disorders — cannot be excluded. The statistically significant baseline mood imbalance (G1 higher than G0, p=0.005) represents a potential source of confounding for mood-related secondary outcomes; although sensitivity analysis demonstrated robustness of the primary conclusion, mood disturbance advantages in G1 should be interpreted with appropriate caution. The study did not collect metabolic parameters (serum lipids, blood pressure, fasting glucose), precluding assessment of potential cardiovascular and metabolic effects associated with E2-containing therapy, which have been highlighted in recent evidence syntheses (22). The sample size resulted in limited power for individual adverse event comparisons. Subgroup analyses by menopausal stage, baseline symptom severity, and age were not pre-specified and would have been substantially underpowered in this sample. Consistency of treatment-associated differences across patient subgroups cannot be confirmed from these data and should be examined in future larger prospective studies. A larger multicentre prospective cohort incorporating metabolic endpoints, breast density measurement, and extended follow-up would be required to comprehensively characterize long-term risk–benefit profiles.

## 5 Conclusion

In this single-center retrospective cohort study, E2/DYD combination therapy was associated with significantly greater short-term perimenopausal symptom reduction compared with dydrogesterone monotherapy, with a clinically meaningful KMI score difference, a higher clinical response rate, and greater improvements in hot flush and mood disturbance symptoms. Endometrial measurements in the combination therapy group showed modest increases within the range reported for E2/DYD in controlled trials, with week-12 mean values remaining below the clinical surveillance threshold; no patient required further endometrial investigation during the observation period. Adverse events were predominantly mild physiological responses to estrogen exposure and did not result in treatment discontinuation. As a retrospective observational analysis, causal conclusions cannot be drawn, and the observed associations may be subject to residual confounding, including confounding by indication. Dydrogesterone monotherapy was associated with meaningful symptom improvement and represents a clinically appropriate option for selected patients in whom estrogen is contraindicated. The 12-week follow-up does not permit conclusions regarding long-term endometrial or breast safety. Prospective randomized trials incorporating metabolic endpoints, breast density assessment, and extended follow-up are required to confirm these associative findings and to characterize long-term benefit–risk profiles.

## Data Availability

The raw data supporting the conclusions of this article will be made available by the authors, without undue reservation.
